# The p36 Isoform of Murine Cytomegalovirus m152 Protein Suffices for Mediating Innate and Adaptive Immune Evasion

**DOI:** 10.3390/v5123171

**Published:** 2013-12-16

**Authors:** Annette Fink, Angeliqué Renzaho, Matthias J. Reddehase, Niels A. W. Lemmermann

**Affiliations:** Institute for Virology, University Medical Center of the Johannes Gutenberg-University Mainz, Obere Zahlbacher Str. 67, Mainz D-55131, Germany; E-Mails: finka@uni-mainz.de (A.F.); renzaho@uni-mainz.de (A.R.)

**Keywords:** antigen presentation, BAC mutagenesis, CD8 T cells, cytomegalovirus, viral immune evasion, natural killer (NK) cells, *N*-linked glycosylation

## Abstract

The MHC-class I (MHC-I)-like viral (MHC-Iv) *m152* gene product of murine cytomegalovirus (mCMV) was the first immune evasion molecule described for a member of the β-subfamily of herpesviruses as a paradigm for analogous functions of human cytomegalovirus proteins. Notably, by interacting with classical MHC-I molecules and with MHC-I-like RAE1 family ligands of the activatory natural killer (NK) cell receptor NKG2D, it inhibits presentation of antigenic peptides to CD8 T cells and the NKG2D-dependent activation of NK cells, respectively, thus simultaneously interfering with adaptive and innate immune recognition of infected cells. Although the *m152* gene product exists in differentially glycosylated isoforms whose individual contributions to immune evasion are unknown, it has entered the scientific literature as m152/gp40, based on the quantitatively most prominent isoform but with no functional justification. By construction of a recombinant mCMV in which all three *N*-glycosylation sites are mutated (N61Q, N208Q, and N241Q), we show here that *N*-linked glycosylation is not essential for functional interaction of the m152 immune evasion protein with either MHC-I or RAE1. These data add an important functional detail to recent structural analysis of the m152/RAE1γ complex that has revealed *N*-glycosylations at positions Asn61 and Asn208 of m152 distant from the m152/RAE1γ interface.

## 1. Introduction

Murine cytomegalovirus (mCMV) encodes a set of MHC class I (MHC-I)-like molecules (MHC-Iv) from the “mCMV-private” *m145* gene family that are involved in evasion of natural killer (NK) cell recognition of infected cells by interfering with cell surface expression of MHC-I-like cellular ligands of the activatory NK cell receptor NKG2D. Specifically, m145 interferes with MULT1 [1], m152 with RAE1 family members [2], and m155 with H60 [3,4] (for reviews, see [5,6,7,8,9]).

Besides representing the first confirmed MHC-Iv type immune evasion molecule of a CMV [10,11,12], m152 is special in that it targets not only RAE1 family ligands of NKG2D for subverting innate immune recognition of infected cells but also classical MHC-I allomorphs for inhibiting the recognition of infected cells by virus epitope-specific CD8 T cells, hence subverting also adaptive immunity ([13,14,15]; for reviews see [16,17,18]).

Mechanistically, regarding its interference with the classical MHC-I pathway of antigen-presentation, m152 is thought to interact transiently with nascent peptide MHC-I complexes (pMHC-I) in the ER and disconnects them from the constitutive vesicular flow to the cell surface by retaining them in the ER-Golgi Intermediate Compartment (ERGIC)/cis-Golgi [11,12], which classifies m152 as the prototype of a “retainer”-type immune evasion molecule (for reviews, see [16,19]). Accordingly, the frequent statement that MHC-I cell surface expression is “downmodulated” by m152 may be somewhat misleading. More precisely, the function of this immune evasion molecule is to interfere with trafficking of newly generated pMHC-I from the ER to the cell surface, while loss of virus-specific as well as overall cell surface pMHC-I rather results from cell-surface MHC-I turnover in absence of resupply [20]. Transient interaction between pMHC-I and the luminal portion of m152, which is a type-I transmembrane protein, proved to be sufficient for catalyzing durable pMHC-I retention, while dissociated m152 passes the Golgi apparatus and eventually becomes degraded in the lysosome [21]. Regarding m152’s interference with cell surface expression of NKG2D ligands of the RAE1 family, the association with m152 varies between different RAE1 isoforms, with the greatest affinity observed for RAE1γ [22]. RAE1δ appears to be special in that its nascent form is effectively retained by m152, whereas loss of the mature, surface-resident form is prevented by absence of a PLWY motif [23]. Based on the high affinity of m152’s interaction with RAE1γ, Wang and colleagues [24] succeeded in resolving the X-ray crystal structure of the m152/RAE1γ complex, and they defined intermolecular contacts showing that m152 interacts in a pincer-like manner with two sites on the α1 and α2 helices of RAE1γ.

In infected cells, m152 is found in differentially glycosylated isoforms, of which a 40 kDa molecular species is most prominent [12]. This has led to equate m152 with gp40 in its immunoevasive functions, both in innate and adaptive immune recognition of infected cells, although the isoform(s) actually interacting with and catalyzing retention of classical MHC-I and RAE1 molecules as well as a possible contribution of carbohydrate moieties to the retention function have never been established. The crystal structure of the m152/RAE1γ complex indeed revealed electron density for two single *N*-acetyl glucosamine residues at Asn61 and Asn208 [24], which shows that the *N*-glycosylation does at least not interfere with the physical association between m152 and RAE1γ. Whether or not it is actually needed for the immunoevasive function is the question that we have pursued here.

## 2. Results and Discussion

### 2.1. Impaired Immune Evasion Function Coincides with Quantitative Underrepresentation of Glycosylation Isoform gp48 of m152

Our original interest in the role of glycosylation isoforms of m152 was based on the incidental observation of inconsistent reduction in overall cell surface display of classical MHC-I molecules in two mCMV mutants deleted for immune evasion gene *m06* [25] but supposed to be identical in the expression of the two remaining mCMV-encoded class-I trafficking regulators m152 and m04, namely mutants mCMV-Δm06^L^ [18,26] and mCMV-Δm06^W^ [27]. Identical expression of m04/gp34 [28] by these two mutants has been documented previously [18], so that suspicion focused on a possibly aberrant expression of m152.

**Figure 1:**
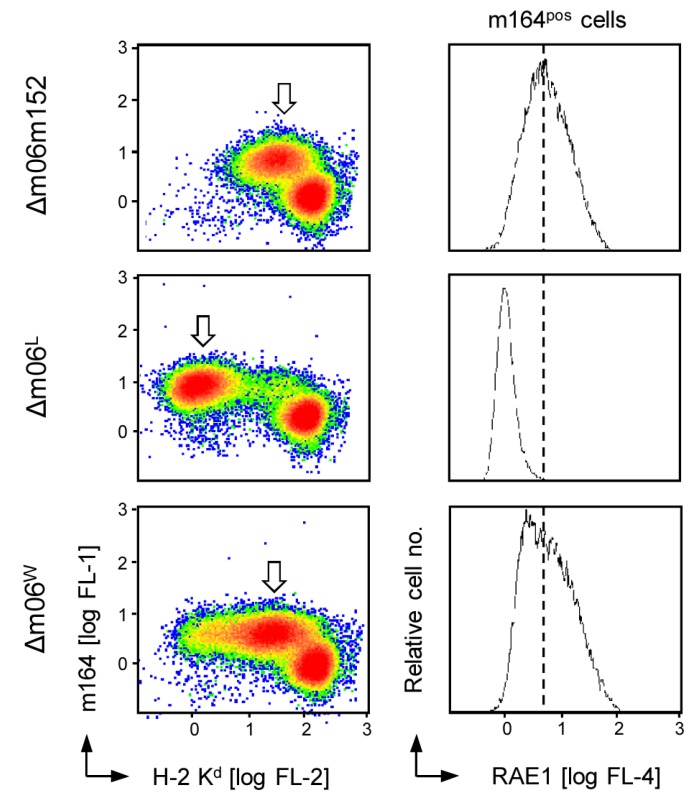
Inconsistent effects of m152 expression in cells infected with Δm06 mutants. *Left column panels*: Cytofluorometric analysis of cell surface MHC-I (H-2 K^d^) expression (abscissa; FL-2, PE fluorescence intensity) and expression of the ER-resident, E-phase infection marker m164/gp36.5 (ordinate; FL-1, Alexa Fluor488 fluorescence intensity) in BALB/c MEF infected with the indicated immune evasion gene deletion mutants of mCMV. Data are displayed as density plots (color-coded with red and blue representing highest and lowest density, respectively). The arrows point to the population of actually infected MEF present within the infected cell cultures beyond to cells that are non-permissive for productive cycle infection and express high levels of MHC-I not downmodulated by immune evasion gene expression. *Right column panels*: Cell surface expression of pan-RAE1 (abscissa; FL-4, APC fluorescence intensity) by the gated population of infected MEF. To serve as a reference for an easier comparison, the dashed line marks the peak RAE1 expression in absence of immune evasion molecules m06 and m152.

**Figure A1:**
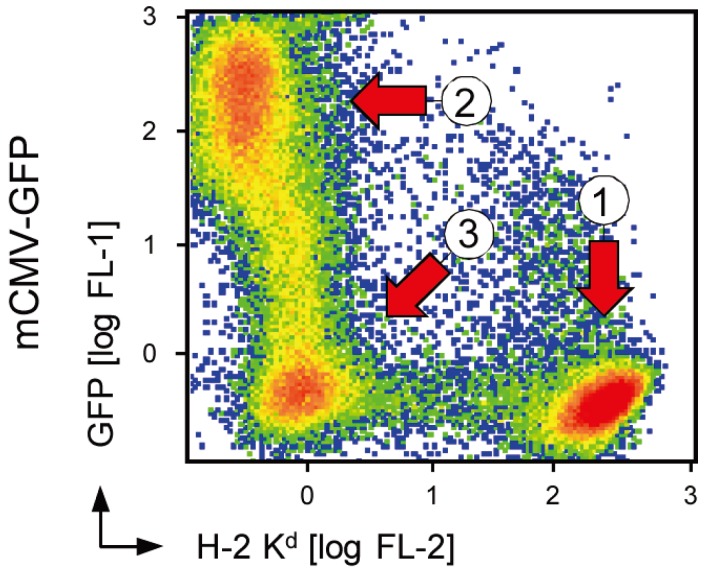
Reporter virus revealing cells non-permissive for the viral replicative cycle within infected mouse embryo fibroblast cell cultures. Cytofluorometric analysis of cell surface MHC-I (H-2 K^d^) expression (abscissa; FL-2, PE fluorescence intensity) and expression of the fluorescent reporter protein GFP (ordinate; FL-1) in BALB/c MEF infected with reporter virus mCMV-GFP. Data are displayed as a density plot (color-coded with red and blue representing highest and lowest density, respectively). The red arrows point to three prominent cell populations. Population 1: Cells not expressing the GFP reporter under control of the human CMV promoter-enhancer but expressing a high level of cell surface MHC-I, which indicates lack of immune evasion gene expression. Population 2: Infected cells expressing high levels of reporter protein GFP but low levels of MHC-1 due to the action of the immune evasion proteins. Population 3: Putatively infected cells, in which GFP expression became silenced and in which MHC-I expression is low due to a preceding action of the immune evasion proteins.

As shown in Figure 1, left column, infected cultures of mouse embryo fibroblasts (MEF) consist of two cell populations with clearcut distinction between “uninfected” cells characterized by missing expression of the ER-resident viral early (E) phase glycoprotein m164/gp36.5 [29] and high cell surface expression of classical MHC-I, and infected cells characterized by expression of the infection marker m164/gp36.5 and levels of MHC-I cell surface expression that vary depending upon the expression of immune evasion genes [18,26,30]. As a side aspect for clarity, it should be noted that at an MOI of 4 all cells present in infected cell cultures likely have virus attached, but apparently not all cells are permissive for the viral gene expression program. Notably, these cells also fail to express a fluorescent reporter, such as GFP, even under the control of the human CMV IE promoter-enhancer in a respective recombinant virus mCMV-GFP [31] (Appendix Figure A1), which suggests immediate silencing of incoming viral genomes.

After infection with virus mCMV-Δm06m152, infected m164^pos^ cells (arrow-marked) showed an intermediate level of cell surface MHC-I, which was strongly reduced by expression of m152 in cells infected with mCMV-Δm06^L^. Surprisingly, although mCMV-Δm06^W^, which is the prototypic m06 deletion mutant used in several publications by a number of investigators, was expected to show the same phenotype, immune evasion was found to be alleviated [30], corresponding to an only moderate reduction of cell surface MHC-I expression (Figure 1, left column). Notably, these findings for the three viruses were paralleled by cell surface levels of RAE1 in the respective m164^pos^ cell populations (Figure 1, right column), which clearly points to m152 expression as being the key to this phenomenon, since RAE1, unlike classical MHC-I, is targeted by m152 selectively.

We therefore studied the expression of m152 by these two m06 deletion mutants in comparison to mCMV-WT.BAC. Generally, as shown by us previously in Western blot analyses, the amount of the 40 kDa molecular species of the m152 protein is strongly reduced in cells infected with mCMV-Δm06^W^ compared to cells infected with WT virus or mCMV-Δm06^L^ [18]. That the protein is not completely absent has been shown by Western blot in the original report on mCMV-Δm06^W^ [27], and we could confirm this by sensitive immunofluorescence analysis of infected cells as well as by immunohistological detection of m152 protein [18] in tissue sections of mice infected with mCMV-Δm06^W^ [32]. 

**Figure 2:**
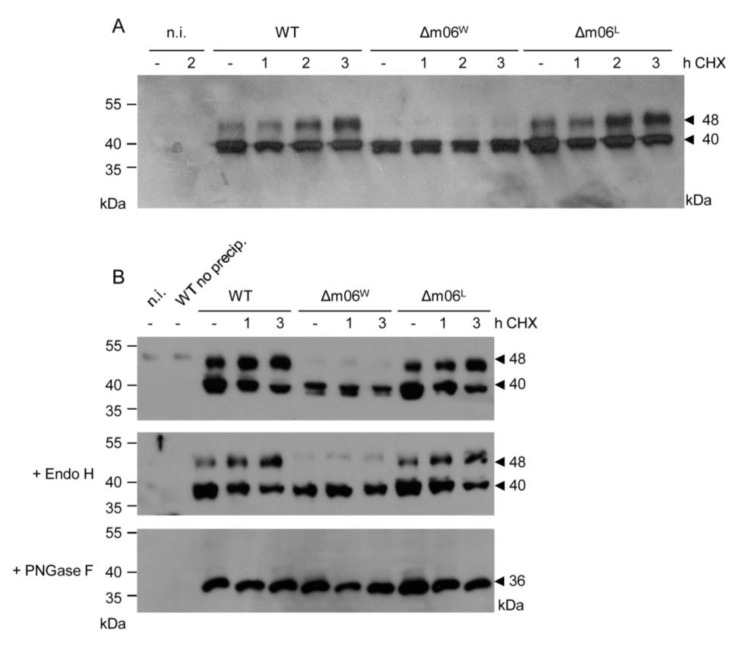
Protein expression and glycosylation analysis of molecular species of m152 after infection of BALB/c MEF with mCMV-Δm06^W^ or -Δm06^L^ compared to mCMV-WT. BAC (WT). MEF were infected with the indicated viruses and, from 6 h p.i. onward, further translation was blocked with cycloheximide (CHX) for the indicated times to reveal shifts to higher glycosylated isoforms over time. To increase the sensitivity of detection by enrichment of m152 isoforms prior to Western blot analysis, 430 µg of total cell protein lysates were subjected to immunoprecipitation (IP) by using magnetic Dynabeads incubated with monoclonal antibody 152.01. (**A**) Molecular identification of m152 isoforms. Solubilized IP-precipitates were subjected to SDS-PAGE (12.5%) and molecular species of m152 were detected by Western blot using monoclonal antibody M3D10; (**B**) Glycosylation analysis**.** Prior to SDS-PAGE and Western blotting, bead-bound IP-precipitates were mock treated (*upper panel*) or were treated with either Endo H (*centre panel*) or PNGase F (*lower panel*) for 60 min n.i., not infected; no precip., IP procedure performed without a precipitating antibody.

Here we have modified the approach of detecting m152 proteins in that they were first enriched by m152-specific immunoprecipitation before their analysis by Western blot (Figure 2). For WT virus and mutant virus mCMV-Δm06^L^, this more sensitive analysis revealed m152 isoforms of 48 kDa and 40 kDa, whereas the 48 kDa molecular species proved to be severely underrepresented selectively in cells infected with virus mCMV-Δm06^W^ (Figure 2A). In accordance with previous work by Ziegler and colleagues describing the more abundant 40 kDa molecular species [21], both isoforms turned out to be resistant against treatment with endoglycosidase H (Endo H), while removal of all *N*-linked carbohydrates using PNGase F identified them as differentially *N*-glycosylated isoforms gp48 and gp40 that, according to their Endo H resistance, must have passed the Golgi apparatus, and led to a deglycosylated (more precisely: *N*-linked carbohydrate-deprived) isoform p36 (Figure 2B). As indicated by increasing abundance of gp48 corresponding to decreasing abundance of gp40 over time in absence of further protein synthesis, gp40 is apparently a precursor of the higher-glycosylated isoform gp48. It should be noted that the reason for the difference between the two Δm06 mutants is still unknown, as sequencing did not reveal any mutation in the coding region, the 3' and 5' untranslated regions (UTRs), and the promoter region of transcription unit m152 of the two Δm06 viruses (data not shown).

Based on these findings we surmised the gp48 glycosylation isoform of m152 might be the functional isoform of m152 in immune evasion. 

### 2.2. Mapping of *N*-glycosylation Sites in m152 and Generation of a Recombinant Virus Expressing only Isoform p36 of the m152 Gene Product

**Figure 3:**
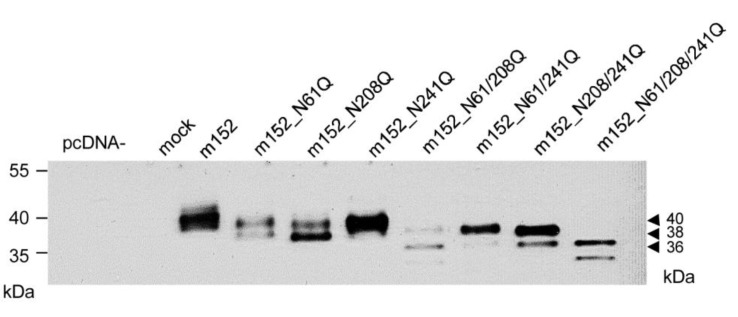
Mutational analysis of *N*-glycosylation sites in m152. Western blot analysis of m152 isoforms (not enriched by a preceding IP) expressed in COS7 cells transfected with expression plasmids carrying single or combined N/Q mutations at potential *N*-glycosylation sites. In essence, COS7 cells were transfected with 4 µg of the indicated expression vectors. After 48 h, total protein extraction was performed and 30 µg of the protein lysates were subjected to SDS-PAGE (12.5%) followed by Western blot analysis using monoclonal antibody M3D10 for detection.

There exist three potential *N*-glycosylation sites in the amino acid sequence of the m152 protein, namely at amino acid positions Asn61, Asn208, and Asn241. Transfection of COS7 cells with expression plasmids carrying single mutations N61Q, N208Q, and N241Q revealed a predicted *ca.* 2 kDa molecular mass shift from 40 kDa to 38 kDa only after mutations N61Q or N208Q (Figure 3), indicating that the site Asn241 is not used in presence of either of the other two sites, at least not in COS7 cells. Interestingly, as mentioned above, the crystal structure of the m152/RAE1γ complex, for which the ectodomain of His6-tagged m152 was expressed in Drosophila S2 cells, in fact revealed usage of both Asn61 and Asn208, but not of Asn241 [24]. In accordance with these structural data, double mutation N61/208Q revealed a molecular species of 36 kDa consistent with p36 found after de-*N*-glycosylation with PNGase F. In line with non-usage of Asn241, mutation of all three sites also revealed p36 as the most prominent band. A minor signal at lower apparent molecular mass turned out to be visible only upon transfection (see below) and, therefore, we did not pursue its identity any further.

**Figure 4:**
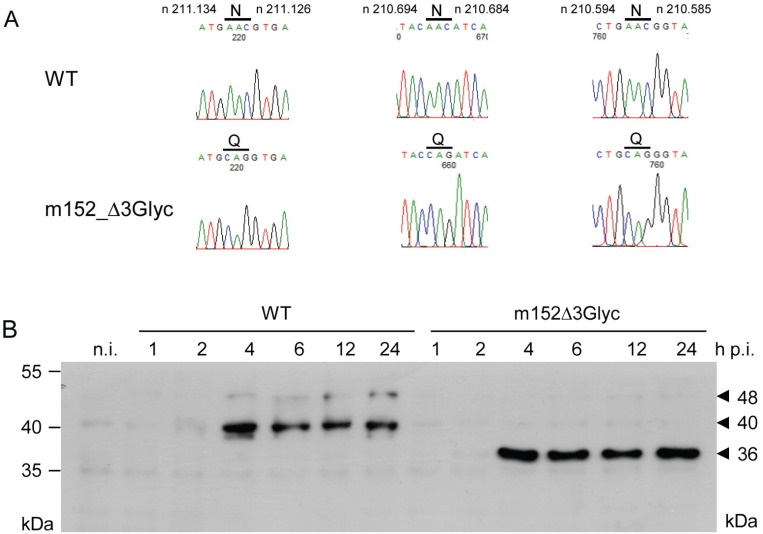
Verification of N/Q mutations in the m152 coding sequence of recombinant BAC plasmid and virus m152Δ3Glyc. (**A**) Chromatograms of BAC plasmid sequencing. *Upper panels:* WT.BAC (WT) sequence regions of interest with Asn (N) at aa positions 61, 208 and 241 (positions defined by protein start). Indicated are nucleotide positions n of the WT.Smith genome [35]. *Lower panels:* m152Δ3Glyc.BAC sequence regions of interest with Gln (Q) at the respective aa positions. Signals from nucleotides A, T, G, and C are shown in green, red, black, and blue, respectively; (**B**) Western blot analysis of m152 protein expression. BALB/c MEF were infected with mCMV-WT.BAC (WT) or mCMV-m152Δ3Glyc. At the indicated times p.i., proteins were extracted and 30 µg of whole protein lysates were subjected to SDS-PAGE (12.5%) followed by Western blot analysis using monoclonal antibody M3D10 for detection of m152 isoforms.

For analyzing any functional impact of N-glycosylation on the immune evasion function of m152, we constructed and characterized mutant virus mCMV-m152Δ3Glyc. Fidelity of triple mutagenesis N61Q, N208Q, and N241Q is verified by sequencing (Figure 4A), and Western blot analysis revealed a single molecular species of 36 kDa, that is the p36 isoform of m152 (Figure 4B). Note that a lower molecular mass species, whose existence was suggested by transfection with the triple-N/Q expression plasmid (see above), is not seen in cells infected with the corresponding virus mCMV-m152Δ3Glyc.

**Figure A2:**
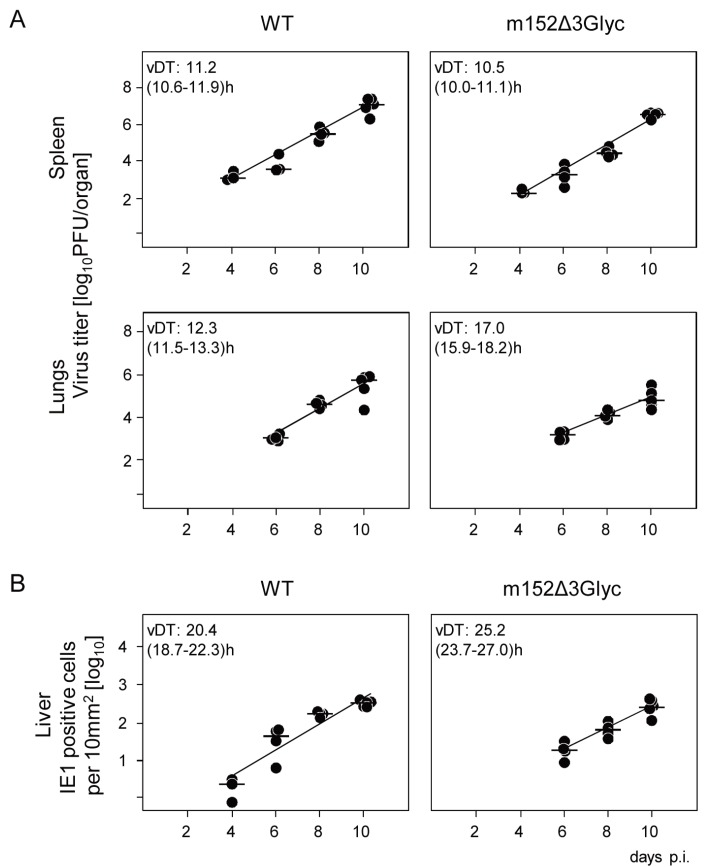
Comparative growth kinetics of mCMV-WT.BAC and mCMV-m152Δ3Glyc in immunocompromised mice. Prior to intraplantar infection with 1 × 10^5^ PFU of the viruses under comparison, BALB/c mice were immunocompromised by hematoablative total-body γ-irradiation with a single dose of 6.5 Gy. At the indicated times p.i., virus multiplication was quantitated in host organs. (**A**) Virus titers (plaque forming units, PFU) in spleen (*upper two panels*) and lungs (*lower two panels*), determined by virus plaque assay under conditions of centrifugal enhancement of infectivity; (**B**) Infection of the liver quantitated by counting the number of infected liver cells, which are mostly hepatocytes, stained by IHC specific for intranuclear IE1 protein. Symbols represent individual mice. Median values are marked by short horizontal bars. Virus doubling times in hours (vDT, with 95% confidence intervals in parentheses) were calculated from the slopes of log-linear regression lines determined by using the statistics software Mathematica [39]. Note that slopes, unlike the absolute ordinate values, reflect virus growth within the respective tissues/organs, independent of possible differences in the spread to these organs and not influenced by unintentional systematic error or statistical variance in inoculum virus doses.

Log-linear growth curves in organs of immunocompromised mice revealed identical, exponential growth of mCMV-WT.BAC and mutant virus mCMV-m152Δ3Glyc in the spleen but suggest some growth attenuation of the mutant virus in lungs and liver as indicated by a somewhat prolonged virus doubling time in these organs (Appendix Figure A2). Although some residual innate immunity from NK cells remains in BALB/c mice immunocompromised by a 6–7 Gy dose of γ-irradiation [33,34], a link of growth attenuation of mCMV-m152Δ3Glyc to a potentially impaired innate immune evasion of NK cell control is considered less likely, as one would not expect this to apply to liver and lungs but not to the spleen where NK cells are, in principle, operative as well. Furthermore, the log-linear growth curves of virus mCMV-Δm152 that activates NK cells through its failure to prevent RAE1 cell surface expression did not reveal an attenuation in liver and lungs (unpublished data), which excludes attenuation of mCMV-m152Δ3Glyc due to NK cell control; hence, the reason for the slight growth attenuation of mCMV-m152Δ3Glyc in liver and lungs remains to be revealed but is most likely unrelated to m152 and its glycosylation status.

### 2.3. The 36 kDa Isoform of m152 Expressed by Virus mCMV-m152Δ3Glyc is Sufficient to Inhibit Antigen Presentation to CD8 T Cells

**Figure 5:**
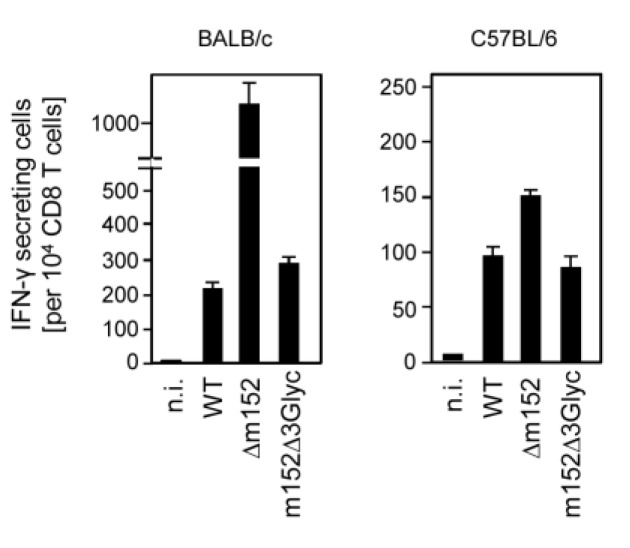
Inhibition of antigen presentation to CD8 T cells by m152 independent of its *N*-glycosylation status. Polyclonal memory CD8 T cells derived from the spleens of latently infected BALB/c (*left panel*) and C57BL/6 (*right panel*) mice at seven months after intraplantar infection with mCMV-WT.BAC were used as effector cells in an IFN-γ ELISpot assay for sensing the presentation of antigenic peptides on BALB/c and C57BL/6 MEF, respectively, infected with the indicated viruses either expressing all isoforms of m152 (WT), lacking m152 (Δm152), or expressing just the unglycosylated p36 isoform (m152Δ3Glyc). *n.i.*, uninfected MEF. Bars represent numbers of responding CD8 T cells, error bars represent 95% confidence intervals.

One function of m152 is to retain peptide-loaded classical MHC-I molecules in the ERGIC, thereby reducing the number of pMHC-I complexes displayed at the cell surface for recognition by CD8 T cells. The presentation of pMHC-I complexes can be evaluated by quantitating the number of virus epitope-specific but polyclonal CD8 T cells sensitized to secrete IFN-γ upon contact with infected cells. As shown in Figure 5 for memory CD8 T cells derived from latently infected BALB/c and C57BL/6 mice representing haplotypes H-2^d^ and H-2^b^, respectively, recognition of WT virus-infected MEF was low compared to cells infected with the immune evasion gene deletion mutant mCMV-Δm152. Importantly, in this respect, the m152 *N*-glycosylation-deprived mutant virus mCMV-m152Δ3Glyc behaved like WT virus, with no noticeable difference in the degree of immune evasion. Thus, N-glycosylation of m152 is not critically involved in the immune evasion function of m152 in antigenic peptide presentation by classical MHC-I molecules of two different MHC haplotypes.

### 2.4. The 36 kDa Isoform of m152 Expressed by Virus mCMV-m152Δ3Glyc is Sufficient to Inhibit Activation of NK Cells through RAE1/NKG2D Interaction

**Figure 6:**
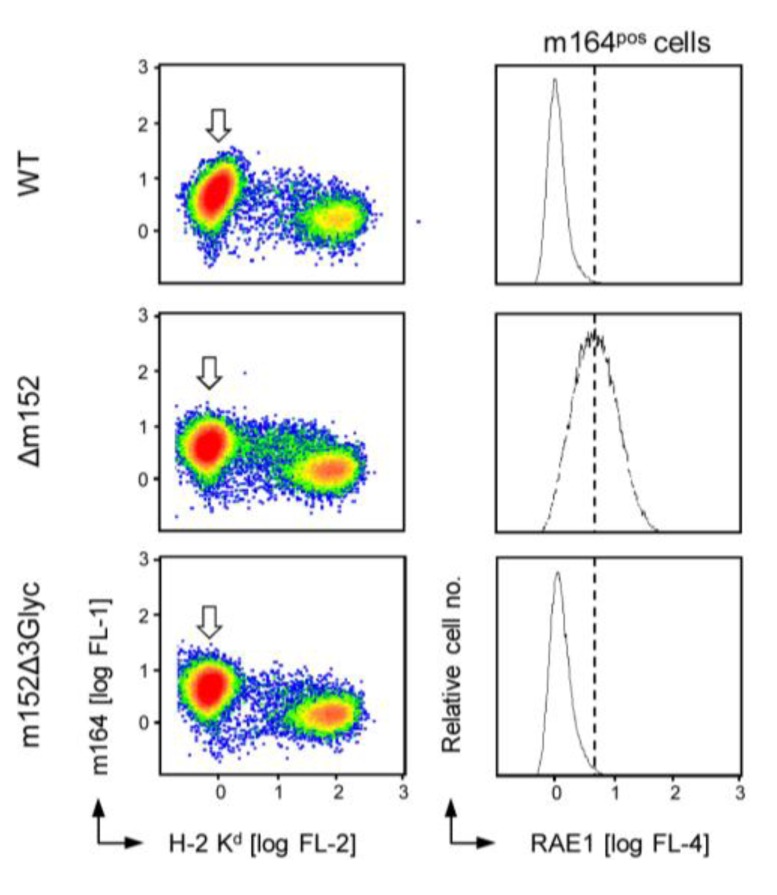
Inhibition of RAE1 cell surface expression by m152 independent of its *N*-glycosylation status. Cytofluorometric analysis of pan-RAE1 cell surface expression (*right column panels:* abscissa; FL-4, APC fluorescence intensity) on BALB/c MEF infected with the indicated viruses either expressing all isoforms of m152 (WT), lacking m152 (Δm152), or expressing just the unglycosylated p36 isoform (m152Δ3Glyc). To serve as a reference for an easier comparison, the dashed line marks the peak RAE1 expression in absence of immune evasion molecule m152. The analysis was restricted to actually infected cells (*left column panels*: arrow-marked population) by electronic gating on cells expressing E-phase infection marker m164/gp36.5 (ordinate; FL-1, Alexa Fluor488 fluorescence intensity) and low levels of MHC-I (H-2 K^d^, abscissa; FL-2, PE fluorescence intensity). For data display, see legend to Figure 1.

**A**lthough it is proposed that the identified crystal structure of the complex formed between m152 and the MHC-I-like NKG2D ligand RAE1 is a paradigm for MHC/MHC interactions, including the interaction of the MHC-I-like m152 molecule with classical MHC-I [24], *N*-glycosylation might nonetheless modulate m152/MHC-I and m152/RAE1 interactions differently and possibly with different functional consequences. We therefore also studied the impact of *N*-glycosylation of m152 on RAE1 cell surface expression by comparing cells infected with mCMV-m152Δ3Glyc with cells infected with WT virus, known to downmodulate cell surface RAE1, and mutant virus mCMV-Δm152, known to leave RAE1 untouched (Figure 6). Again, analysis was restricted to infected (arrow-marked) cells characterized by expression of the infection marker m164/gp36.5 and low cell surface expression of classical MHC-I that, in the case of these three viruses (and different to the viruses used in Figure 1), was mediated primarily by immune evasion protein m06, which is known to be the main regulator of overall cell surface MHC-I expression [18,26,27]. Importantly, m152Δ3Glyc, that is the m152 isoform p36, proved to be fully competent in downmodulating cell surface RAE1.

**Figure 7:**
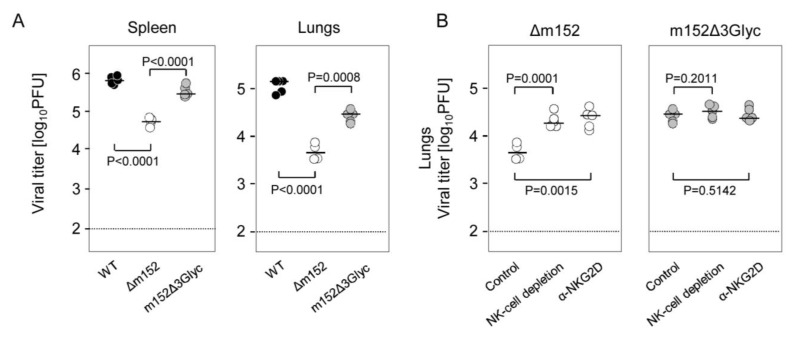
NK-cell evasion by m152 independent of its *N*-glycosylation status. (**A**) Modulation of the *in vivo* NK cell control of virus replication by m152 expression in spleen (*left panel*) and lungs (*right panel*) of mice infected with viruses expressing all isoforms of m152 (WT, black-filled circles), lacking m152 (Δm152, open circles), or expressing just the p36 isoform (m152Δ3Glyc, gray-filled circles); (**B**) Replication of mCMV-m152Δ3Glyc in the lungs is not influenced by NK cells. Left panel: Growth attenuation of virus mCMV-Δm152 dependent upon activation of NK cells through ligation of the activatory receptor NKG2D. Right panel: NK cell-independent growth of mCMV‑m152Δ3Glyc. The day-3 NK cell assay was performed with BALB/c mice left undepleted (control), BALB/c mice depleted of pan-NK cells with anti-asialo GM-1 antibodies administered 24 h before infection, or BALB/c mice in which ligation of NKG2D on NK cells was blocked by antagonistic anti-NKG2D antibodies administered 6 h before infection. Throughout, symbols represent virus titers (determined as plaque-forming units, PFU, by virus plaque assay performed under conditions of centrifugal enhancement of infectivity) for individual BALB/c mice infected i.v. three days before with 2 × 10^5^ PFU of the indicated viruses. Median values are marked by short horizontal bars. The dotted lines indicate the detection limit. The statistical significance of differences was tested based on log-transformed ordinate values by the unpaired, two-tailed Student’s t test with Welch’s correction not assuming equal variance. Differences are considered non-significant for *p* > 0.05, significant for *p* < 0.01, and highly significant for *p* < 0.001.

The effects on RAE1 cell surface expression were functionally reflected *in vivo* by the antiviral activity of NK cells in spleen and lungs in an established 3-day NK cell assay (for a review, see [6]) (Figure 7A). Specifically, NK cell control of virus replication, which is effective after infection with the m152 deletion virus mCMV-Δm152, was significantly alleviated in BALB/c mice infected with either the WT virus (*p* < 0.0001) or the mutant virus mCMV-m152Δ3Glyc (*p* < 0.0001). The somewhat lower replication of mCMV-m152Δ3Glyc compared to WT virus in the lungs likely relates to its slight growth attenuation already seen in Appendix Figure A2 for the lungs of immunocompromised mice (see the discussion above). This definitively does not indicate a reduced NKG2D ligation-specific NK cell evasion of virus mCMV-m152Δ3Glyc since, in contrast to mCMV-Δm152, its replication in the lungs was not increased by pan-NK cell depletion or by antagonistic anti-NKG2D antibody blocking the activatory RAE1/NKG2D interaction (Figure 7B).

In conclusion, N-glycosylation of m152 does not appear to be critical—and is definitively not essential—for m152/RAE1 interaction that prevents NKG2D-dependent activation of NK cells.

## 3. Experimental

### 3.1. Cells, Viruses, and Mice

Primary BALB/c or C57BL/6 mouse embryo fibroblasts (MEF) were cultivated in modified Eagle’s medium (MEM) supplemented with 10% fetal calf serum (FCS) and antibiotics. For transfection experiments, COS7 cells were cultivated and seeded 24 h prior to transfection in DMEM medium supplemented with 10% FCS and antibiotics.

Virus derived from BAC plasmid pSM3fr [36] was used as “wild-type” virus, mCMV-WT.BAC (WT). Recombinant viruses mCMV-Δm06^W^ and mCMV-Δm06^L^ are described in references [26] and [27], respectively. Virus mCMV-GFP has been described by Angulo and colleagues [31].

BALB/c mice were bred and maintained under SPF conditions at the “Central Laboratory Animal Facility (CLAF)” of the University Medical Center Mainz. All experimental procedures were performed in compliance with the ‘International Guiding Principles for Biomedical Research Involving Animals’ guidelines. The experiments were approved according to German federal law under permission number AZ 1.5 177-07-04/051-61.

### 3.2. Infection Conditions and Virus Growth Kinetics in Immunocompromised Mice

For *in vitro* assays, 3rd-passage MEF were infected with the indicated viruses at a multiplicity of infection (MOI) of 4 by using the method of centrifugal enhancement of infectivity ([37,38] and references therein).

For the *in vivo* NK cell assay (see below), 8–10 week-old BALB/c mice were infected intravenously (i.v.) with 2 × 10^5^ PFU (non-enhanced) of mCMV-WT.BAC or the indicated BAC-derived recombinant viruses, all diluted in 50 µL of PBS.

Priming of mice for the generation of memory CD8 T cells was performed by intraplantar infection with 1 × 10^5^ PFU (non-enhanced) of mCMV-WT.BAC.

Log-linear *in vivo* virus growth curves were determined by intraplantar infection (see above) of BALB/c mice immunocompromised by hematoablative treatment with a single 6.5 Gy dose of total-body γ-irradiation and subsequent monitoring of organ infection on days 2, 4, 6, 8, and 10. Infection of lungs and spleen was quantitated by determining virus titers (see above) in the respective organ homogenates. Infection of the liver was quantitated by immunohistochemistry (IHC) and counting of infected liver cells, which are predominantly hepatocytes, in representative 10 mm^2^ areas of tissue sections, based on detection of the intranuclear immediate-early (IE) protein IE1-pp89/76. Virus doubling times (vDT) and their 95% confidence intervals (CI) were calculated from the slopes of regression lines determined by linear regression analysis with the statistics software Mathematica [39]. All methods were described in greater detail previously [38].

### 3.3. *In Vivo* NK Cell Assays

On Day 3 after i.v. infection (see Section 3.2), virus titers in homogenates of spleen and lungs were determined under conditions of centrifugal enhancement of infectivity. The involvement of NK cells was tested by NK-cell depletion with polyclonal rabbit antibody directed against mouse asialo GM1 (20 μL in 500 μL of PBS i.v., WAKO Chemicals, Richmond, VA, USA) administered on day-1, *ca.* 24 h before infection. For the specific blockade of NK-cell activation through RAE1/NKG2D ligation, hamster monoclonal antibody (clone C7) directed against mouse NKG2D [40] was administered i.v. (300 μg in 500 μL of PBS) 6 h before infection.

### 3.4. ELISpot Analysis

An IFN-γ-based enzyme-linked immunospot (ELISpot) assay was performed to quantify responding memory CD8 T cells after stimulation with infected MEF. Polyclonal memory CD8 T cells were immunomagnetically purified from spleen cell populations of latently infected mice at 7 months after primary intraplantar infection with mCMV-WT.BAC. The assay has been described in detail previously ([41] and references therein). In brief, graded numbers of CD8 T cells were incubated in triplicate cultures for 18 h with 1 × 10^5^ MEF infected with an MOI of 4 (centrifugal infection) for 90 min prior to the cocultivation during which infection proceeds. Frequencies of IFN-γ-secreting cells and the corresponding 95% confidence limits were calculated from the spot counts by intercept-free linear regression analysis with the statistics software Mathematica [39].

### 3.5. Cytofluorometric Analysis

At 16 h p.i. (MOI 4, with centrifugal enhancement), BALB/c MEF were stained for cell surface MHC-I with PE-conjugated monoclonal antibody mouse anti-mouse H-2 K^d^ (FL-2, clone SF1-1.1; BD Pharmingen, catalog no. 553566, Heidelberg, Germany), for cell surface RAE1 with APC-labeled monoclonal antibody rat anti-mouse pan-RAE1 (FL-4, clone 186107; R&D Systems, catalog no. FAB17582A; Wiesbaden, Germany), and for intracellular expression of m164/gp36.5 indirectly with a rabbit anti-m164 antiserum [30] and Alexa Fluor488-conjugated goat-anti-rabbit IgG (FL-1, Life technologies, catalog no. A11008; Darmstadt, Germany). GFP fluorescence was measured in fluorescence channel 1 (FL-1). Analysis was performed with a Beckman Coulter FC500 cytofluorometer and CXP software [42].

### 3.6. Construction of Expression Plasmids

To generate expression plasmids, PCR was performed using mCMV-WT.BAC genomic DNA with oligonucleotides m152-HpaI_for/rev (Appendix Table A1) amplifying the full length ORF*m152* with the following protocol parameters: 5 min at 95 °C; 18 cycles of 30 s at 94 °C, 90 s at 62 °C, and 2 min at 68 °C; 12 cycles of 30 s at 94 °C, 2 min at 45 °C and 2 min at 68 °C; followed by final extension for 10 min at 68 °C. The PCR product was subcloned into vector pcDNA3.1 within the *EcoR* I restriction site. To insert nucleotide exchanges CAT-GCG at nt positions 211.131–211.129, 210.690–210.688, and 210.591–210.589, the construct was subjected to site-directed mutagenesis using the Quick Change II Site-Directed Mutagenesis Kit (Agilent, catalog No. 200524, Böblingen, Germany) with the oligonucleotide pairs m152_N61Q_for/_rev, m152_N208Q_for/_rev, and m152_N241Q_for/rev (Appendix Table 1), respectively. The mutagenesis resulted in constructs pcDNA-m152N61Q, pcDNA-m152N208Q, and pcDNA-m152N241Q. Plasmid pcDNA-m152N61Q was subjected to a second round of mutagenesis using the respective oligonucleotides resulting in the constructs pcDNA-m152N61/208Q and pcDNA-m152N61/241Q. Plasmid pcDNA-m152N208Q was used as template for the construction of pcDNA-m152N208/241Q, and, in a third round, mutagenesis of pcDNA-m152N61/208Q resulted in the generation of pcDNA-m152N61/208/241Q (m152Δ3Glyc). The successful replacement was confirmed by sequencing (GATC; Konstanz, Germany).

### 3.7. Generation of Recombinant Viruses

To generate recombinant virus mCMV-m152Δ3Glyc, BAC mutagenesis was performed. In a first step, mCMV-WT.BAC DNA was used as template for PCR with the oligonucleotides m152_BAC-for/rev (Appendix Table A1) to amplify ORF*m152* with the following protocol parameters: 5 min at 95 °C; 35 cycles of 15 s at 94 °C, 1 min at 55 °C and 7 min 40 s at 68 °C; followed by final extension for 10 min at 72 °C. After *Sac* I/*Xma* I restriction, the product was subcloned into pBluescript, resulting in pB-m152_flank. The m152_208/241 fragment from pcDNA-m152N208/241Q (see above) was inserted into pB-m152_flank by *Nhe* I/*Sph* I subcloning. The thus modified m152 sequence was inserted into the shuttle vector pST76K_SR by *Sac* I/*Xma* I restriction, resulting in pST76K-m152_N208/241Q_flank. This construct was used for allelic exchange of ORF*m152* in BAC plasmid pSM3fr as described [43], resulting in BAC-m152_N208/241. 

For replacement of the third glycosylation site, BAC-m152_N208/241 DNA was introduced into *E. coli* GS1783 and recombination was performed by Red-mediated markerless DNA recombination as described by Tischer and colleagues [44]. In brief, oligonucleotides pEPKan-S_m152N61Q_for/rev (Appendix Table A1) were used for a PCR with plasmid pEP-kanS as a template. The resulting products were transformed into GS1783 cells carrying BAC-m152_N208/241Q. After Red-recombination, arabinose-induced *I-Sce*I expression, and a second round of Red-recombination, m152_N61/208/241Q (m152Δ3Glyc) BAC DNA was purified and successful mutagenesis was confirmed by sequencing (GATC; Konstanz, Germany).

The generation of recombinant BAC plasmid pSM3fr_Δm152 was performed in *E. coli* strain SW105 as described by Warming and colleagues [45] by PCR-based deletion of ORF*m152* between the nucleotides 211,378 und 210,245 [35]. For this, a kanR cassette flanked by homologous viral sequences was amplified from plasmid pKD46 using oligonucleotides m152_del_for/rev (Appendix Table A1) and inserted into the viral genome by ET-recombination. The kanR cassette was subsequently excised by FLP recombinase [45].

Virus reconstitution and purification of a high titer virus stock were performed as described [38].

### 3.8. Protein Extraction and Analysis

MEF were seeded in 10 cm cell culture dishes and infected with an MOI of 4 under conditions of centrifugal enhancement of infectivity. At defined times p.i., cells were washed with ice-cold PBS and scraped-off. After centrifugation (5 min, 3,000 rpm, 4 °C), the cells in the pellet were lysed for 15 min on ice in 200 µL Lysis Buffer/dish (0.2 M NaCl, 1.5 mM MgCl, 4 mM EDTA, 4 mM EGTA, 1% Triton-X100, 20 mM HEPES; with complete proteinase inhibitor (diluted 1:25; Roche, catalog No.11697498001) and 1mM DTT added shortly before use). After centrifugation (10 min, 14,000 rpm, 4 °C), supernatants were collected and the amount of protein was determined by BCA-Assay (Thermo Scientific, catalog No. 23225, Dreieich, Germany). 30 µg of total protein was separated on an SDS-PAGE followed by Western blot analysis. Isoforms of m152 were detected with monoclonal antibody M3D10 (1:250) [21].

### 3.9. Transfection

5 × 10^5^ COS7 cells per 10-cm dish were seeded and 4 µg DNA was transfected with Polyfect (Qiagen, catalog No. 301105, Hilden, Germany) following the manufacturer’s instructions. 48 h later, cells were harvested and total protein was extracted. 30 µg of protein lysates were separated on a 12.5% SDS-PAGE followed by Western Blot analysis.

### 3.10. Immunoprecipitation

MEF were infected at an MOI of 4 (centrifugal enhancement) and translation was blocked by cycloheximide (CHX) (100 µg/mL) from 6 h p.i. onward. At the indicated times after addition of CHX, protein extraction was performed and 430 µg of protein lysates were incubated overnight with anti-m152 monoclonal antibody (clone 152.01; 1:100)-pretreated Dynabeads (Life technologies, catalog no. 11201D, Darmstadt, Germany). Unbound proteins were removed by repetitive washing with PBS. Precipitated m152 protein isoforms were identified by SDS-PAGE separation and Western blot analysis. 

### 3.11. Deglycosylation with Endo H and PNGase F

Immunoprecipitated, bead-bound m152 isoforms were mock treated or incubated with either 750U EndoH (NEB, catalog no. P0702S, Frankfurt, Germany) or 1000U PNGase F (NEB, catalog No. P0704S, Frankfurt, Germany) for 60 min at 37 °C, followed by SDS-PAGE separation and Western blot analysis.

## 4. Conclusions

The recently established crystal structure of the m152/RAE1γ complex has revealed two single *N*-acetyl glucosamine residues at Asn61 and Asn208 of m152 [24]. As these two positions are distant from the identified m152/RAE1γ interface, Wang and colleagues proposed that these *N*-glycosylations are unlikely to affect that interaction. Here we show functional data confirming this view experimentally. In a transfection system with single mutations of the three potential *N*-glycosylation sites of m152, namely Asn61, Asn208, and Asn241, evidence of usage of the respective site was provided only for Asn61 and Asn208, precisely the two sites actually found to be *N*-glycosylated in the crystallized m152/RAE1γ complexes. Lack of m152 *N*-glycosylation in an N61Q-N208Q-(N241Q) triple mutated virus mCMV-m152Δ3Glyc did not prevent downmodulation of RAE1 from the surface of infected cells, indicating that *N*-glycosylation at Asn61 and Asn208 is not needed for m152/RAE1γ complex formation nor does it alter the complex in a mode relevant for its function in NK cell evasion. Interestingly, the same also applied to the interaction between m152 and classical MHC-I in the inhibition of pMHC-I cell surface trafficking for antigen presentation to CD8 T cells. This supports the title notion by Wang and colleagues that the structural basis of m152 interaction with RAE1γ may reveal a paradigm for MHC/MHC interaction in immune evasion [24].

In addition, these data have shown that absence of the higher glycosylated isoform gp48 of m152 has no functional consequence in immune evasion and thus cannot explain the impaired immune evasion capacity of virus mutant mCMV-Δm06^W^ compared to mCMV-Δm06^L^_._ Since the Asn61/208 dually *N*-glycosylated isoform gp40 is Endo H-resistant, it must have reached the Golgi apparatus, whereas pMHC-I complexes and RAE1 stick in the ERGIC/cis-Golgi. Unless we assume retrograde trafficking of gp40 back into the ER, for which there exists no evidence and for which there is no rationale as glycosylation is not needed for function, the Endo H-resistant isoform gp40 is unlikely the molecule that mediates immune evasion by interacting with and catalyzing the retention of pMHC-I and RAE1. Since *N*-glycosylation takes place in the ER, it remains open to question if nascent p36 interacts with pMHC-I and RAE-1 before or after the glycosylations at Asn61 and Asn208. In either case, our data have revealed that unglycosylated p36 can interact and that the two *N*-glycosylations are not required for the innate and adaptive immune evasion functions of m152.

## Appendices

**Table A1:** Oligonucleotides used in this study; nucleotides inserting aa substitutions are highlighted in bold face.

Name	Sequence
m152-HpaI_for	5'-GGAGTTAACCATATAAAAGCTGTCCCCCATGCCATTCGATCAGACGCGGGCTACTCCCGAAAGAGTAAC-3'
m152-HpaI-rev	5'-GGAGTTAACTGACTAATAAGTTATCTTTATTGTACAAGTGTTGTGTGTTATCCCTGAGCCCATTTTCAG-3'
m152_N_83_Q_for	5'-CATTTTCCTTGGATG**CAG**GTGAGCGAGCTGG-3'
m152_N_83_Q_rev	5'-CCAGCTCGCTCAC**CTG**CATCCAAGGAAAATG-3'
m152_N_230_Q_for	5'-GGTTCCGTTGGCGTAC**CAG**ATCAGTCTCGCGAACGG-3'
m152_N_230_Q_rev	5'-CCGTTCGCGAGACTGAT**CTG**GTACGCCAACGGAACC-3'
m152_N_230_Q_for	5'-GGTTCCGTTGGCGTAC**CAG**ATCAGTCTCGCGAACGG-3'
m152_N_263_Q_rev	5'-CGCGAAGTCGGTACTACC**CTG**CAGGTCTCTGAGATCGAGC-3'
m152_BAC_for	5'-GCGAGTTCGTCTCGAAG-3'
m152_BAC_rev	5'-TAGACCGCCGACAATCAG-3'
pEPKan-S_m152N83Q_for	5'-CTTTCGGATCGAAGCCTCCGGAGAGGTGAAACATTTTCCTTGGATG**CAG**GTGAGCGAGCTGGCGCAGCAACCAATTAACCAATTCTGATTAG-3'
pEPKan-S_m152N83Q_rev	5'-ACGAAGAACGCACTCTCCTGCGCCAGCTCGCTCAC**CTG**CATCCAAGGAAAATGTTTCACCAGGATGACGACGATAAGTAGGG-3'
m152_del_fwd	5'-TGTCACCGCTCCACGTTTCACCGTCGGTCTCCCGATCGCTAGCCTGTACACAGGAACACTTAACGGCTGA-3'
m152_del_rev	5'-GAGCACCCGACGATCTGACATTGTCCAGTGTGCCGGTCGCACGAACATCAAGGACGACGACGACAAGTAA-3'
